# Antimicrobial Activities of Propolis Nanoparticles in Combination with Ampicillin Sodium Against Methicillin-Resistant *Staphylococcus aureus*

**DOI:** 10.3390/microorganisms13081844

**Published:** 2025-08-07

**Authors:** Kaiyue Feng, He Sang, Han Jin, Peng Song, Wei Xu, Hongzhuan Xuan, Fei Wang

**Affiliations:** 1 School of Pharmaceutical Sciences and Food Engineering, Liaocheng University, Liaocheng 252059, China; 2 College of Agriculture and Biology, Liaocheng University, Liaocheng 252059, China; 3 Shandong Key Laboratory of Applied Technology for Protein and Peptide Drugs, Liaocheng University, Liaocheng 252059, China

**Keywords:** methicillin-resistant *Staphylococcus aureus*, propolis nanoparticles, ampicillin sodium, synergy, biofilm

## Abstract

Combining antibiotics with propolis is an effective method to combat bacterial drug resistance. Nanoparticles are of interest in the antimicrobial field because of their higher drug stability, solubility, penetration power, and treatment efficacy. In this study, propolis nanoparticles (PNPs) were synthesized, and their antibacterial and anti-biofilm activities against methicillin-resistant *Staphylococcus aureus* (MRSA) in combination with ampicillin sodium (AS) were analyzed. The PNPs had an average particle diameter of 118.0 nm, a polydispersity index of 0.129, and a zeta potential of −28.2 mV. The fractional inhibitory concentration indices of PNPs and AS against tested MRSA strains highlighted this synergy, ranging between 0.375 and 0.5. Crystal violet staining showed that combined PNPs and AS significantly inhibited biofilm formation and reduced existing biofilm biomass. We then discovered that PNPs inhibited bacterial adhesion, extracellular polysaccharide synthesis, and *mecR1*, *mecA*, *blaZ,* and *icaADBC* gene expression. These results indicated that PNPs exerted a synergistic antibacterial effect with AS by inhibiting *mecR1*, *mecA*, and *blaZ* gene expressions to reduce the drug resistance of MRSA. Meanwhile, PNPs weakened bacterial adhesion and aggregation by suppressing *icaADBC* gene expression, allowing antibiotics to penetrate the biofilm, and exhibiting significant synergistic anti-biofilm activity. In summary, PNPs are promising candidates for combating MRSA-related diseases.

## 1. Introduction

The escalating misuse of antibiotics has driven the emergence of multidrug-resistant bacterial pathogens at an alarming rate, leading to a global increase in serious infectious diseases [1]. Methicillin-resistant *Staphylococcus aureus* (MRSA) is a highly pathogenic multidrug-resistant bacterium that can cause a variety of human diseases such as pneumonia, septicemia, and soft tissue infections [2]. MRSA significantly contributes to hospital-acquired infections, resulting in increased mortality rates, prolonged hospital stays, and elevated public health expenditures [3]. It is one of the most prevalent infectious complications during the initial postoperative year following heart transplantation, underscoring its clinical impact in immunocompromised populations [4]. Through expressing penicillin-binding proteins 2a (PBP2a) and β-lactamases, MRSA is highly resistant to β-lactam antibiotics such as ampicillin [5,6]. It continues to develop new resistance mechanisms with clinical exposure, resulting in varying degrees of resistance to antibiotics such as aminoglycosides, tetracyclines, and macrolides. These developments, combined with increasing detection rates, show that MRSA is becoming an increasingly serious public health problem [7,8].

In addition to antibiotic resistance in planktonic cells, biofilm formation is a pressing issue in the treatment of MRSA. A biofilm is a structurally organized bacterial community encased within a self-synthesized matrix of extracellular polymeric substances, which adheres to both biotic and abiotic surfaces. These substances consist mainly of extracellular polysaccharides along with extracellular proteins and DNA [9]. Studies suggest that approximately 80% of bacterial infections are associated with biofilm formation, with major-antibiotic-treated patients experiencing persistent recurrences because of biofilm resistance [10]. Compared with planktonic bacteria, bacteria encapsulated in biofilms are more resistant to antibiotics and more difficult to remove [11]. Their resistance to conventional antibiotics can be 10–1000 times greater than that of planktonic bacteria [12]. Biofilms allow bacteria to effectively adhere to surfaces and protect them from the host immune system and antibiotic clearance, increasing bacterial pathogenicity and further limiting antibiotic efficacy [13]. Infections caused by biofilm-associated pathogens are difficult to eradicate and can progress from acute to chronic stages, leading to serious complications. Biofilm-associated infections can lead to delayed wound healing, tissue necrosis, and systemic infections, and can cause cross-infection between hospitalized patients [14,15]. MRSA is recognized as one of the predominant causative agents in biofilm-related infections, and developing new drugs to target MRSA biofilms is urgently needed.

One promising strategy is to combine natural products with antibiotics to harness synergistic antibacterial effects, reducing side effects and overcoming the challenges of drug resistance. Propolis is a natural product that has long been used in folk medicine, made by mixing the resin collected by bees with their secretions. It has been shown to have antibacterial, antiviral, antifungal, and antitumor activities [16,17]. In recent years, nanoparticles have shown promise in pharmaceutical and medical applications because of their unique physical and chemical properties, such as their ultra-small size, high surface-area-to-mass ratio, and enhanced chemical reactivity [18]. The use of propolis nanoparticles (PNPs) in the antimicrobial field is of increasing interest because of their higher drug stability, solubility, penetration power, and treatment efficacy [19,20]. However, research on the synergistic antibacterial effect of PNPs and antibiotics, as well as their effects on biofilms, is lacking. This study constructed propolis nanoparticles and evaluated their anti-MRSA and -biofilm activities in combination with antibiotics, elucidating the mechanisms involved.

## 2. Materials and Methods

### 2.1. Propolis Material and Bacterial Strains

Propolis samples were collected in 2023 from Xuancheng City in Anhui Province, China, with poplars (*Populus* sp.) as the predominant plant origin. Methicillin-resistant *S. aureus* (MRSA) ATCC 43300, ATCC 33591, and clinical isolates CI2 and CI3 tested in this study were maintained in our laboratory (the Natural Product Bioactivity Laboratory in the School of Pharmaceutical Sciences and Food Engineering of Liaocheng University). Strains were stored in trypticase soy broth (TSB) medium containing 20% glycerol at −80 °C.

### 2.2. Preparation of PNPs

Propolis samples were mechanically fragmented into fine particles. The fragmented material was immersed in 95% ethanol at a ratio of 1:10 (*w*/*v*), followed by incubation at 37 °C for 48 h on a shaker at 150 rpm. The ethanolic mixture was filtered through Whatman No. 4 filter paper. To remove wax interference, the filtrate was subjected to phase separation at 4 °C for 24 h, followed by secondary filtration through Whatman No. 1 filter paper. The filtrate was concentrated using a rotary evaporator (YRE-2000B, Gongyi, China) until it reached a constant weight [18,21].

For the preparation of PNPs, the prepared extract was dispersed in the aqueous phase and homogenized approximately using a low-temperature ultra-high-pressure continuous-flow cell disruptor (JN-Mini Pro, JNBIO. Guangzhou, China). The concentration of the dispersed PNPs was adjusted to 512 μg/mL. The homogenization process was carried out under a controlled low-temperature environment of −4 °C, with an operating pressure of about 1500 bar, and the total number of cycles was 3. To obtain dried PNPs, a freeze dryer (SCIENTZ-10 N/C, Ningbo, China) was used.

### 2.3. Determination of the Characteristics of PNPs

The characterization of PNPs was carried out according to previously described methods [18,22]. To determine the particle size distribution and polydispersity index (PDI) of the synthesized PNPs, dynamic light scattering (DLS) measurement tests were carried out. Additionally, the zeta potential of the PNPs was determined at 25 °C using a Nano Laser Particle Size Analyzer (Zetasizer Nano ZS, Malvern, UK).

### 2.4. Determination of Minimal Inhibitory Concentration (MIC)

The minimal inhibitory concentration (MIC) of PNPs and ampicillin sodium (AS) against MRSA was determined by the broth microdilution method [23]. Briefly, the tested MRSA strains (ATCC 43300, ATCC 33591, CI2, and CI3) were pre-incubated in TSB medium to the logarithmic growth phase. Cells were collected and diluted to 3 × 10^6^ colony-forming units (CFU)/mL using TSB medium. Subsequently, 100 μL of the samples at different concentrations was mixed with the same volume of bacterial suspension in 96-well microplates, resulting in final sample concentrations of 8–256 μg/mL. The mixed suspension was incubated at 37 °C for 18 h. Subsequently, 20 μL of resazurin sodium (1 mg/mL) was dispensed into all wells and maintained in darkness at 37 °C for 3 h. The MIC was defined as the lowest concentration of PNPs that did not cause the solution to change color from purple to orange.

### 2.5. Determination of Fractional Inhibitory Concentration Indices (FICIs)

The fractional inhibitory concentration indices (FICIs) were evaluated using the checkerboard method [24]. In brief, the tested MRSA strains (ATCC 43300, ATCC 33591, CI2, and CI3) were pre-activated and the bacterial concentrations were adjusted to 3 × 10^6^ CFU/mL. In 96-well plates, serial dilutions of PNPs starting at 2 MIC were performed along the horizontal axis of the plate; serial dilutions starting at 2 MIC were performed along the vertical axis for AS. Next, equal volumes of the MRSA suspensions were added to each well. After incubation at 37 °C for 18 h, the FICIs of the PNPs and AS were measured. The interactions between PNPs and AS were then evaluated by the FICI, calculated as follows: FICI = FIC A + FIC B, where FIC A is MIC_PNPs_ in combination/MIC_PNPs_ alone and FIC B is MIC_AS_ in combination/MIC_AS_ alone. The FICI was interpreted as follows: FICI ≤ 0.5, synergy; 0.5 < FIC ≤ 1.0, additive effect; 1.0 < FICI ≤ 4.0, no effect; and FICI > 4.0, antagonism.

### 2.6. Effect of PNPs in Combination with AS on MRSA Biofilm Formation

The anti-biofilm-formation assay was performed according to published reports [8,25]. Briefly, MRSA cells were cultured and the bacterial density was modified to 3 × 10^7^ CFU/mL. Next, 100 μL of the MRSA bacterial suspensions (ATCC 43300, ATCC 33591, CI2, and CI3) was exposed to equal volumes of PNPs, AS, and PNPs in combination with AS. The final concentrations of PNPs and AS alone against the above strains were 32, 32, 64, and 32 μg/mL and 16, 8, 32, and 32 μg/mL, respectively. Additionally, combinations of 32, 32, 64, and 32 μg/mL of PNPs and 16, 8, 32, and 32 μg/mL of AS were used in 96-well microplates. A quantity of sucrose was added to every well in the plates, resulting in a final concentration of 1%. The plates were then incubated at 37 °C for 24 h. After cultivation, the planktonic bacterial cells were removed and each well was washed three times with phosphate-buffered saline (pH 7.4). In accordance with the literature, the crystal violet (CV) assay was used to determine the biomass of the MRSA biofilm [26].

### 2.7. Effect of PNPs in Combination with AS on Mature MRSA Biofilm

Two hundred microliters of bacterial suspensions of MRSA (ATCC 43300, ATCC 33591, CI2, and CI3) containing 1% sucrose (1.5 × 10^7^ CFU/mL) was dispensed into each well. The samples were incubated at 37 °C for 24 h to form mature biofilms. Planktonic cells were then removed and washed three times. Next, 200 μL of fresh TSB with PNPs (32, 32, 64, and 32 μg/mL) alone, AS (16, 8, 32, and 32 μg/mL) alone, and a combination of PNPs (32, 32, 64, and 32 μg/mL) and AS (16, 8, 32, and 32 μg/mL) was added into each well and cultured again. Simultaneously, every well in the plate was supplied with 1% sucrose. MRSA biofilm biomass was also measured using CV assays, as described in the literature [26].

### 2.8. Determination of Bacterial Adhesion

The impact of PNPs on MRSA adhesion was evaluated using an established protocol [27]. First, 100 μL of bacterial suspension (3 × 10^8^ CFU/mL) of MRSA ATCC 43300 was added to the wells of a 96-well microplate. Subsequently, an equal volume of PNPs with sucrose was introduced into each well; the final concentration of PNPs ranged from 32 to 128 μg/mL, and the final sucrose concentration was maintained at 1%. TSB containing 1% sucrose was employed as the control. Following incubation at 37 °C for various durations (2, 4, 6, 8, 10, and 12 h) to enable cell attachment, planktonic cells were removed and all wells were then washed three times with phosphate-buffered saline. Next, 200 μL of TSB was added to each well, with the wells then exposed to ultrasound for 5 min. Finally, the optical density at 600 nm of the wells was measured using a microplate reader (FlexA-200, Aosheng, Hangzhou, China).

### 2.9. Extracellular Polysaccharide Production Analysis

The influence of PNPs on the synthesis of extracellular polysaccharides by MRSA was investigated using an established method [8]. Briefly, an MRSA ATCC 43300 suspension with a final concentration of 1.5 × 10^8^ CFU/mL was treated with varying concentrations of PNPs (32–128 μg/mL). The mixtures were then brought to 1% sucrose and incubated at 37 °C for 24 h. Upon completion of incubation, the combined suspensions were centrifuged at 12,000× *g* for 30 min at 4 °C. The supernatant was carefully removed, and the remaining precipitate was resuspended in sterile water and mixed uniformly. This suspension underwent further centrifugation to extract the water-soluble polysaccharides present in the supernatant.

The precipitates obtained in the final stage were dispersed in 0.1 mol/L NaOH, and centrifuged at 12,000× *g* for 30 min at 4 °C. The resulting supernatant was collected, and three times its volume of 95% ethanol was added to the combined supernatants. These mixtures were then stored at 4 °C overnight. Following this incubation period, another centrifugation step was performed to isolate the alkali-soluble polysaccharide precipitate. Extracellular polysaccharide content was determined using the phenol/sulfuric acid method as previously reported [28].

### 2.10. Real-Time Quantitative PCR Analysis to Determine Gene Expression Levels

The effects of PNPs on the expression of PBP2a protein synthesis and regulatory genes (*mecA*, *mecR1*) in MRSA, as well as a β-lactamase synthesis gene (*blaZ*) and PIA (the major extracellular polysaccharide of MRSA) synthesis-related genes (*icaADBC*), were detected by real-time quantitative PCR (RT-qPCR) [29]. Briefly, the bacterial suspensions of MRSA ATCC 43300 were centrifuged at 12,000× *g* for 30 min at 4 °C, after which the precipitates were collected following incubation with 32 μg/mL PNPs at 37 °C for 24 h. Total RNA extraction was carried out using the flying shark plus bacteria RNA Kit (Nobelab, Jinan, China). The RNA was converted into cDNA with ALL-in-One First-Strand Synthesis MasterMix (LABLEAD, Beijing, China) following the manufacturer’s instructions. The cDNA templates were amplified with 2 × Realab Green PCR Fast Mixture (LABLEAD) through RT-qPCR, performed on QuantStudio (Applied Biosystems, Waltham, MA, USA). Gene expression levels were quantified using the 2(−ΔΔCT) method, with 16S rDNA serving as the reference gene for cycle threshold value normalization [29,30]. Primer sequences can be found in Table 1 [29,31].

### 2.11. Statistical Analysis

Three independent replicates were performed for each experiment. Results are presented as the mean ± standard deviation. Statistical significance was assessed by Student’s *t*-test and one-way analysis of variance in GraphPad Prism 10, with *p* < 0.05 considered significant.

## 3. Results

### 3.1. Characterization of PNPs

**Figure 1:**
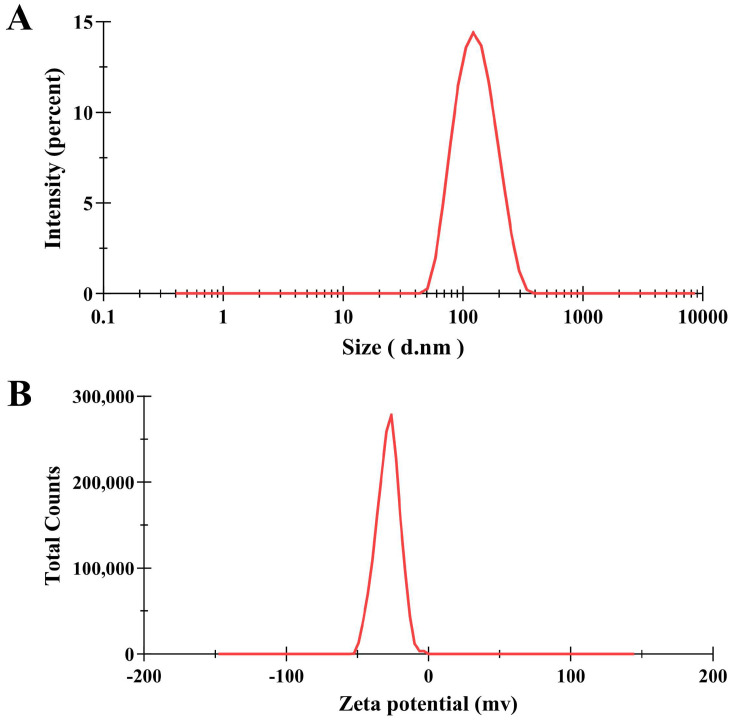
Particle size distribution and potential curve of PNPs. (**A**) Size distribution based on the intensity of PNPs; (**B**) zeta potential of PNPs.

To assess the particle size distribution and PDI, the synthesized PNPs were characterized with DLS. The hydrodynamic diameter of PNPs at 25 °C is presented in Table 2 and Figure 1A, with an average particle diameter of 118.0 nm. A PDI of 0.129 indicated that the synthesized PNPs had a narrow distribution with good homogeneity (Table 2).

The zeta potential, representing the surface electrostatic potential, plays a critical role in determining nanoparticle stability through its dependence on surface charge characteristics and local environmental conditions. The PNPs’ zeta potential indicated an average negative surface charge value of −28.2 mV (Table 2, Figure 1B), which was sufficiently high to avoid nanoparticle aggregation. This indicated that the PNP suspension was kinetically stable without rapid clustering.

### 3.2. MIC of PNPs and AS

The MICs of the samples against all strains were determined by broth microdilution. The MICs of PNPs against MRSA ATCC 43300, ATCC 33591, CI2, and CI3 were 128, 128, 256, and 128 μg/mL, respectively, whereas those of AS were 128, 32, 128, and 128 μg/mL (Table 3). These results indicated that PNPs alone had significant inhibitory activity against the strains, whereas they showed resistance to AS.

### 3.3. Synergistic Effect of PNPs with AS Against Planktonic MRSA

**Figure 2:**
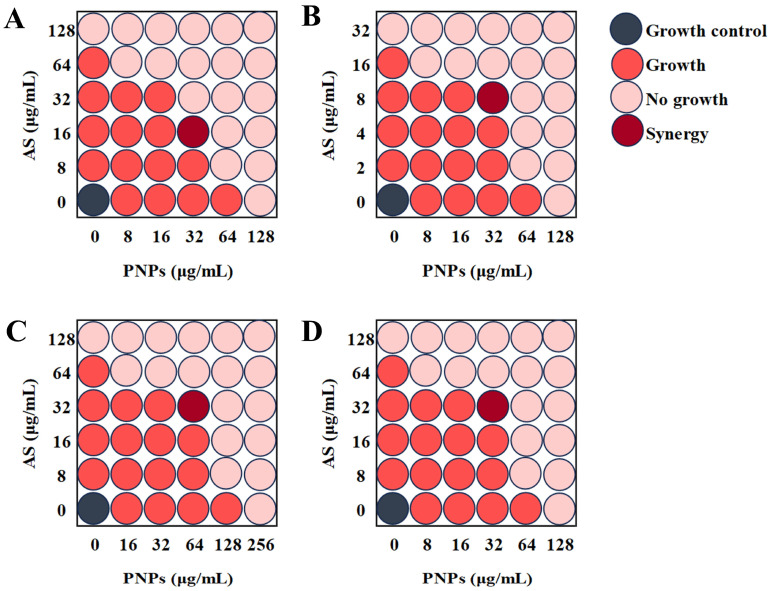
Synergistic effect of AS with PNPs. (**A**) Schematic checkerboard of MRSA ATCC 43300 growth inhibition with varying concentrations of PNPs and AS. (**B**) Schematic checkerboard of MRSA ATCC 33591 growth inhibition with varying concentrations of PNPs and AS. (**C**) Schematic checkerboard of MRSA CI2 growth inhibition with varying concentrations of PNPs and AS. (**D**) Schematic checkerboard of MRSA CI3 growth inhibition with varying concentrations of PNPs and AS.

The FICIs of PNPs in combination with AS against MRSA ATCC 43300, ATCC 33591, CI2, and CI3 were 0.375, 0.5, 0.5, and 0.5, respectively (Table 3, Figure 2). The combination of PNPs and AS had significant synergistic effects on all tested strains, indicating that PNPs may have significant inhibitory effects on MRSA drug resistance mechanisms.

### 3.4. Synergistic Effect of PNPs with AS Against MRSA Biofilm

**Figure 3:**
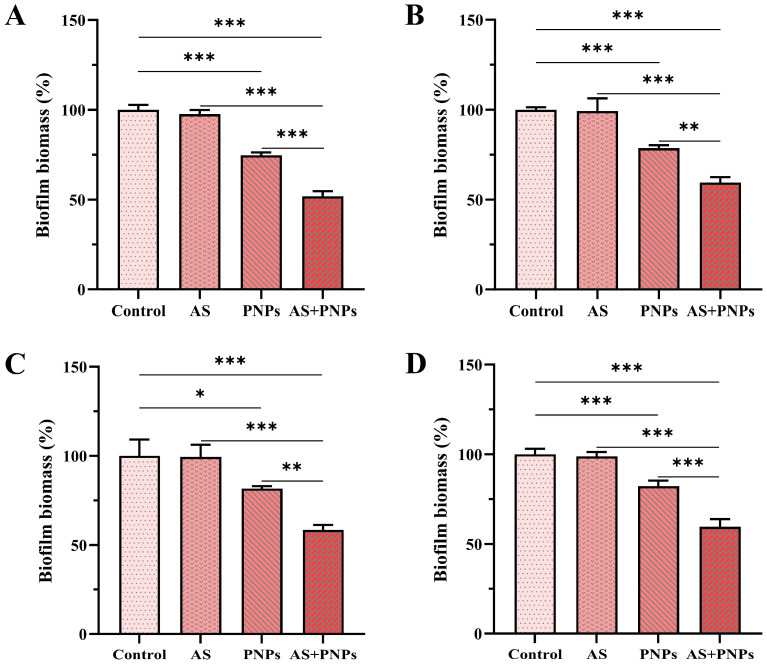
Effects of different samples on MRSA biofilm formation: (**A**) 16 μg/mL of AS, 32 μg/mL of PNPs, and the combination AS + PNPs against MRSA ATCC 43300; (**B**) 8 μg/mL of AS, 32 μg/mL of PNPs, and the combination AS + PNPs against MRSA ATCC 33591; (**C**) 32 μg/mL of AS, 64 μg/mL of PNPs, and the combination AS + PNPs against MRSA CI2; (**D**) 32 μg/mL of AS, 32 μg/mL of PNPs, and the combination AS + PNPs against MRSA CI3 (* *p* < 0.05, ** *p* < 0.01, *** *p* < 0.001).

**Figure 4:**
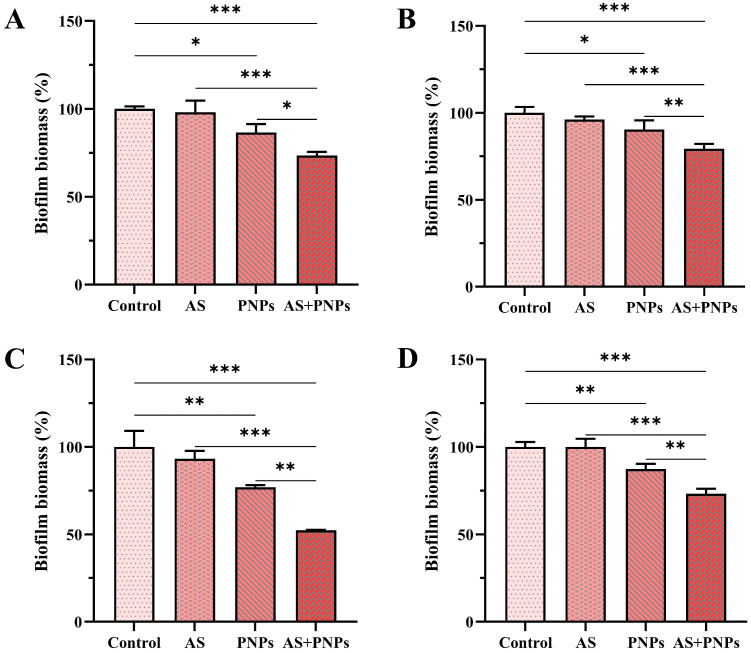
Effects of different samples on mature MRSA biofilm: (**A**) 16 μg/mL of AS, 32 μg/mL of PNPs, and the combination AS + PNPs against MRSA ATCC 43300; (**B**) 8 μg/mL of AS, 32 μg/mL of PNPs, and the combination AS + PNPs against MRSA ATCC 33591; (**C**) 32 μg/mL of AS, 64 μg/mL of PNPs, and the combination AS + PNPs against MRSA CI2; (**D**) 32 μg/mL of AS, 32 μg/mL of PNPs, and the combination AS + PNPs against MRSA CI3 (* *p* < 0.05, ** *p* < 0.01, *** *p* < 0.001).

The total biofilm biomass of MRSA was determined using CV assays. In both the biofilm formation and mature biofilm models, AS alone had no anti-biofilm effect, whereas PNPs alone had inhibitory effects (Figure 3 and Figure 4). The percentage reduction was calculated with the biofilm biomass of the control group as the baseline. For MRSA ATCC 43300, MRSA ATCC 33591, MRSA CI2, and MRSA CI3, in the biofilm formation models, the biofilm biomass in the PNP-alone treatment group was reduced by approximately 25.3%, 21.4%, 18.4%, and 17.8%, and the combined use of AS and PNPs resulted in a reduction in biofilm biomass by approximately 48.2%, 40.4%, 41.5%, and 40.5%, respectively. In mature biofilm models, the biofilm biomass in the PNP-alone treatment group was reduced by approximately 13.5%, 9.7%, 23.1%, and 12.7%, and the combined use of AS and PNPs resulted in a reduction in biofilm biomass by approximately 26.5%, 20.7%, 47.7%, and 26.8%, respectively. Among all MRSA strains detected, the biofilm biomass was significantly reduced in the treatment where PNPs and AS were used in combination compared with the use of PNPs alone, indicating synergistic functions in anti-biofilm efficacy. Low PNP concentrations inhibited biofilms, allowing antibiotics to penetrate to inhibit bacteria and enhance the anti-biofilm effect, suggesting that PNPs may inhibit MRSA adhesion and aggregation.

### 3.5. Effect of PNPs on Bacterial Adhesion

**Figure 5:**
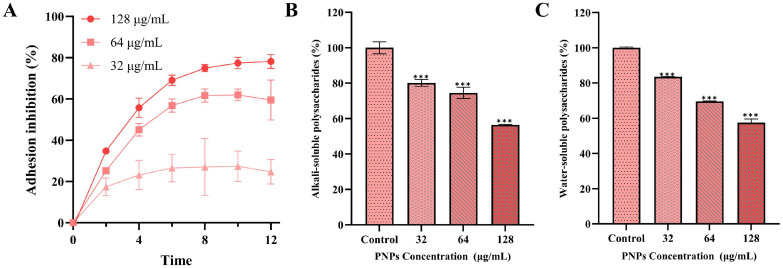
Effects of different concentrations of PNPs on bacterial adhesion and extracellular polysaccharide production of MRSA ATCC 43300. (**A**) PNPs inhibit bacterial adhesion; (**B**) PNPs inhibit alkali-soluble polysaccharide production; (**C**) PNPs inhibit water-soluble polysaccharide production (*** *p* < 0.001).

Bacterial adhesion is a key step in the formation of fresh biofilms and the consolidation of mature biofilms, and strong adhesion gives the biofilm a dense structure that blocks the invasion of antibiotics; in contrast, loose biofilms are unable to prevent antibacterial agent penetration [8]. The adhesion inhibition rate of PNPs at low concentrations of 32 µg/mL on MRSA ATCC 43300 was significant (Figure 5A), increasing significantly with PNP concentration. The results indicated that PNPs could significantly inhibit the adhesion of MRSA, decreasing biofilm compactness and allowing antibiotics to penetrate and exert their synergistic effects with PNPs. The combination of PNPs and AS showed a significant inhibitory effect on MRSA biofilms.

### 3.6. Effect of PNPs on Extracellular Polysaccharide Production

Alkali-soluble polysaccharides play a key role in MRSA adhesion; the adhesive force they provide is critical for the formation of biofilms and the consolidation of mature biofilms. Water-soluble polysaccharides also provide a nutrient source for colonizing pathogens [27,32]. There was an exponential reduction in both alkali- and water-soluble polysaccharide concentrations following PNP treatment at doses ranging from 32 to 128 µg/mL (Figure 5B,C), with their degree of reduction being dose-dependent with PNP concentration. The results showed that after treatment with 32 μg/mg PNPs, the reduction rate of alkali-soluble polysaccharides produced by MRSA was 19.9%, and that of water-soluble polysaccharides was 16.5%. After treatment with 64 μg/mg PNPs, the reduction rate of alkali-soluble polysaccharides produced by MRSA was 25.5%, and that of water-soluble polysaccharides was 30.5%. After treatment with 128 μg/mg PNPs, the reduction rate of alkali-soluble polysaccharides produced by MRSA was 43.5%, and that of water-soluble polysaccharides was 42.6%. These results provide evidence that PNPs effectively suppress polysaccharide biosynthesis in MRSA.

### 3.7. Effect of PNPs on Gene Expression

**Figure 6:**
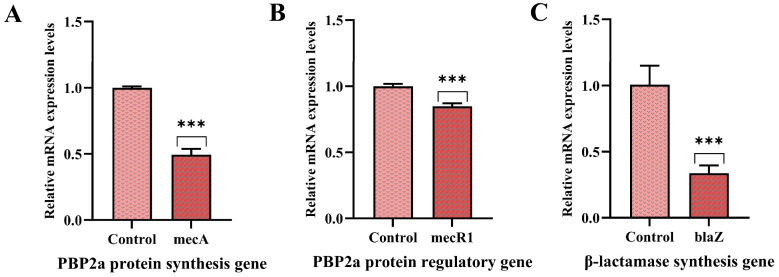
Effects of PNPs on the expression of the *mecA*, *mecR1*, and *blaZ* genes of MRSA ATCC 43300. (**A**) PNPs inhibit the expression of *mecA*; (**B**) PNPs inhibit the expression of *mecR1*; (**C**) PNPs inhibit the expression of *blaZ* (*** *p* < 0.001).

**Figure 7:**
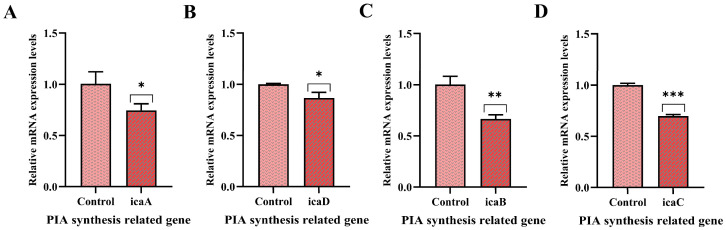
Effects of PNPs on the expression of the *icaADBC* genes of MRSA ATCC 43300. (**A**) PNPs inhibit the expression of *icaA*; (**B**) PNPs inhibit the expression of *icaD*; (**C**) PNPs inhibit the expression of *icaB*; (**D**) PNPs inhibit the expression of *icaC* (* *p* < 0.05, ** *p* < 0.01, *** *p* < 0.001).

The effects of PNPs on the expression of PBP2a protein synthesis and regulatory genes (*mecA*, *mecR1*) in MRSA ATCC 43300, as well as a β-lactamase synthesis gene (*blaZ*) and PIA synthesis-related genes (*icaADBC*), were analyzed. The expression levels of *mecA*, *mecR1*, and *blaZ* were significantly downregulated compared with the control group, suggesting that PNPs substantially inhibit antibiotic resistance in MRSA (Figure 6). Downregulation of gene expression of the PIA-related *icaADBC* was also observed compared with the control group (Figure 7). These results indicated that PNPs not only impaired the drug resistance of planktonic MRSA but also inhibited the formation and consolidation of biofilms by suppressing PIA generation.

## 4. Discussion

In recent years, excessive use of antibiotics has led to a gradual increase in MRSA drug resistance, making the diseases it causes more difficult to treat; this has made it one of the greatest threats to modern medicine [33]. MRSA has become a global problem as it has developed resistance to almost all available β-lactam drugs. Additionally, it can acquire resistance to various alternative antibacterial drugs, resulting in varying degrees of resistance to multiple types of antibiotics and making the infections it causes increasingly difficult to cure [34]. The detection and fatality rates of MRSA are constantly rising, while the effectiveness of antibiotics is dropping significantly. There is an urgent need for new antibacterial drugs or methods to enable existing antibiotics to regain their effectiveness.

Propolis is a biologically rich natural product that performs well as an antimicrobial. Earlier research demonstrated that propolis exhibits significant antimicrobial effects against various pathogenic microorganisms, such as *S. aureus*, *Bacillus subtilis*, *Streptococcus mutans*, and *Escherichia coli* [24,35,36]. Compared with propolis, PNPs have more advantages, such as better drug stability, penetration, and solubility. PNPs have also been studied to some extent for their antibacterial properties. Afrasiabi et al. reported the synergistic efficacy of PNPs in enhancing antibacterial photodynamic therapy against *S. mutans*, and Parolia et al. demonstrated a significant inhibitory effect of PNPs on *Enterococcus faecalis* [18,19]. However, there remains a lack of studies investigating the synergistic effects of PNPs in combination with β-lactam antibiotics against MRSA and its biofilms.

In this study, we successfully synthesized PNPs derived from Chinese propolis. When these nanoparticles were combined with AS, they exhibited a significant inhibitory effect not only on the planktonic cells but also on the formation and consolidation of MRSA biofilms. These findings underscore the potential of PNPs as promising candidates for both the prevention and treatment of diseases associated with MRSA.

The reversal of the main resistance mechanism is the key behind PNPs’ ability to synergistically inhibit MRSA in combination with AS. MRSA primarily develops resistance to β-lactam antibiotics through the production of resistance-related proteins such as PBP2a, which is encoded by the *mecA* gene; its regulator, *mecR1*, senses β-lactam antibiotics to initiate *mecA* expression. This protein acts as a transpeptidase, facilitating bacterial cell wall synthesis even in the presence of β-lactam antibiotics [31,37]. Additionally, the secretion of β-lactamase encoded by *blaZ* can hydrolyze β-lactam antibiotics [31,37]. PNPs were able to significantly downregulate *mecA* and *blaZ* expression, indicating that they could significantly inhibit the expression of PBP2a and β-lactamase at the transcriptional level to reduce cell resistance to β-lactam antibiotics and demonstrating a significant synergistic effect with AS.

The formation of MRSA biofilm further enhances its antibiotic resistance. The development of biofilms follows a dynamic progression through four distinct stages: adhesion, aggregation and proliferation, maturation, and shedding and dissemination. The adhesion and aggregation of microbial cells play a pivotal role in biofilm dynamics, promoting the formation of new biofilms as well as the consolidation of established ones [38,39]. For MRSA, PIA (the major extracellular polysaccharide in biofilms) is significantly positively correlated with its adhesion, indicating that it is a key determinant regulating biofilm formation. Our study showed that PNPs could significantly inhibit the production of alkali- and water-soluble polysaccharides, decreasing biofilm compactness to allow antibiotics to penetrate. This led to the observed significant inhibitory effect of the combination of PNPs and AS on MRSA biofilm.

PIA is catalytically synthesized by proteins encoded by the *ica* operon, which contains four functional genes (*icaADBC*) and one repressor gene (*icaR*). The *icaA*-encoded protein exhibits N-acetylglucosamine transferase activity, and the *icaD* product significantly enhances this enzymatic function [40]. The *icaB* gene encodes a secreted deacetylase, while that encoded by *icaC* is a kind of membrane protein which is responsible for the transport of PIA to the extracellular region [41]. The transcriptional direction of *icaR* is opposite to that of *icaADBC*, and its encoded protein negatively regulates the operon. PNPs significantly downregulated *icaADBC* expression, inhibiting the synthesis of PIA at the transcriptional level and weakening the adhesion ability of MRSA.

Based on these results, we found that PNPs had two functions: they reversed the drug resistance of MRSA by reducing the expression of *mecA* and *blaZ* genes, having a significant synergistic antibacterial effect with AS on planktonic MRSA bacteria; additionally, they reduced the expression of *icaADBC*, decreasing PIA synthesis to decrease biofilm compactness, allowing antibiotic penetration. These findings indicate that PNPs have good potential for use in the field of anti-MRSA infection.

## 5. Conclusions

We synthesized and characterized PNPs, systematically investigating their antibacterial and anti-biofilm activities with AS against MRSA. The combination of PNPs and AS inhibited planktonic MRSA cells and demonstrated significant synergistic effects on biofilms. The significant synergistic antibacterial effect on planktonic MRSA was attributed to the downregulation of *mecR1*, *mecA*, and *blaZ* gene expression by PNPs, increasing AS effectiveness. In biofilms, PNPs weakened bacterial adhesion and aggregation by suppressing the expression of *icaADBC*, enabling antibiotics to penetrate, exhibiting significant anti-biofilm synergism with AS. Overall, PNPs can be considered promising candidates for combating MRSA-related diseases.

## Figures and Tables

**Table 1:** Primers used in this study.

Gene	Primer Sequences (5′→3′)	Reference
*mecA*	F: TCCAGGAATGCAGAAAGACC	[29]
	R: TTGGAACGATGCCTATCTCA	
*mecR1*	F: GTGCTCGTCTCCACGTTAATTCCA	[31]
	R: GACTAACCGAAGAAGTCGTGTCAG	
*blaZ*	F: GCTTTAAAAGAACTTATTGAGGCTTC	[31]
	R: CCACCGATYTCKTTTATAATTT	
*icaA*	F: CTTGCTGGCGCAGTCAATAC	[29]
	R: GTAGCCAACGTCGACAACTG	
*icaD*	F: TGGGCATTTTCGCGATTATCA	[29]
	R: ACGATTCTCTTCCTTTCTGCCA	
*icaB*	F: CCTGTAAGCACACTGGATGG	[29]
	R: TCGCTTTTCTTACACGGTGA	
*icaC*	F: TGCGTTAGCAAATGGAGACT	[29]
	R: TGCGTGCAAATACCCAAGAT	
*16S rRNA*	F: CGCAATGGGCGAAAGC	[29]
	R: TACGATCCGAAGACCTTCATCA	

**Table 2:** The hydrodynamic diameter, PDI, and zeta potential of PNPs at 25 °C.

PNPs	
Size (nm)	118.0 ± 0.40
PDI	0.129 ± 0.01
Zeta potential (mV)	−28.2 ± 0.26

**Table 3:** The individual and combinatorial effects of PNPs and AS.

Bacteria	Combinations	MIC (μg/mL) ^a^	MIC (μg/mL) ^b^	FICI	Result
MRSA ATCC 43300	PNPs	128	32	0.375	Synergy
	AS	128	16		
MRSA ATCC 33591	PNPs	128	32	0.5	Synergy
	AS	32	8		
MRSA CI2	PNPs	256	64	0.5	Synergy
	AS	128	32		
MRSA CI3	PNPs	128	32	0.5	Synergy
	AS	128	32		

MIC ^a^—values of individual samples. MIC ^b^—values of the samples in combination.

## Data Availability

The original contributions presented in this study are included in the article. Further inquiries can be directed to the corresponding author.

## Abbreviations

The following abbreviations are used in this manuscript:

PNPs	Propolis Nanoparticles
MRSA	Methicillin-Resistant *Staphylococcus aureus*
AS	Ampicillin Sodium
PBP2a	Penicillin-Binding Proteins 2a
PIA	Polysaccharide Intercellular Adhesin
TSB	Trypticase Soy Broth
PDI	Polydispersity Index
DLS	Dynamic Light Scattering
MIC	Minimal Inhibitory Concentration
CFU	Colony-Forming Units
FICIs	Fractional Inhibitory Concentration Indices
CV	Crystal Violet
